# Electronically Temperature-Dependent Interplay between He and Trivacancy in Tungsten Plasma-Facing Materials

**DOI:** 10.3390/ma17102182

**Published:** 2024-05-07

**Authors:** Zhao-Zhong Fu, Bi-Cai Pan

**Affiliations:** 1 Key Laboratory of Strongly Coupled Quantum Matter Physics, Department of Physics, University of Science and Technology of China, Hefei 230026, China; fuzhaozh@mail.ustc.edu.cn; 2 Hefei National Laboratory for Physical Sciences at Microscale, University of Science and Technology of China, Hefei 230026, China

**Keywords:** trivacancy, He atom, W plasma-facing materials, electronic structures, tight-binding potential model

## Abstract

Both microvoids and helium (He) impurities are widely present in tungsten (W) plasma-facing materials (PFMs), where the interaction between microvoids and He atoms has led to the intriguing development of microvoids. In this paper, we comprehensively investigated the interaction between He atoms and trivacancy (V3), a fundamental microvoid in W-PFMs, at the level of tight-binding theory. Our study showed that He atoms can catalyze the decomposition of the original V3 or facilitate its transformation into another V3 variant. We propose that a He atom near the V3 defect induces significant changes in the distribution of d-electron charges within the W atoms lining the inner wall of the V3 defect, making the W atom nearest to this He atom cationic and the other W atoms anionic. The attractive interaction between them promotes the decomposition and deformation of V3. As electronic excitation increases, the ionization of W atoms on the V3 wall gradually intensifies, thereby enhancing the cationic characteristics of the W atoms closest to the He atom. This process also prompts other W atoms to shift from anions to cations, leading to a transition in the electrostatic interactions between them from attraction to repulsion. This transformation, driven by electronic excitation, plays a significant inhibitory role in the decomposition and deformation of V3.

## 1. Introduction

During the operation of a nuclear fusion reactor, high-energy neutrons and helium (He) are produced, some of which collide with various components [1,2,3], such as W-PFMs and tritium breeding blankets, creating defects [4,5]. Of various concerns, the interaction of He with different irradiation defects in W-PFMs causes intriguing physical phenomena [6], which has received much attention. Density functional theory (DFT) calculations have shown that an individual He atom in perfect body-centered cubic (BCC) W prefers to occupy tetrahedral interstitial sites (TISs) rather than octahedral interstitial sites (OISs) [7]. Once monovacancies exist in BCC W, a He atom is preferably trapped by a monovacancy due to the lower charge density within the vacancy [8]. As the number of He atoms increases, multiple He atoms are trapped by a single vacancy, promoting the nucleation and growth of He bubbles [7]. In cases where these processes occur near the surface region of the W-PFM, nanosized fuzzy structures may form on the surface [9,10,11,12]. Furthermore, the scenario involving divacancy (V2) has also been investigated. Typically, DFT calculations predicted three distinct configurations for divacancies, and their interactions with hydrogen (H) or helium (He) atoms were assessed [13]. These efforts showed that the binding energy of these three configurations shifts from negative to positive upon introducing a single H atom within each of them, stabilizing these V2 structures. However, introducing a He atom yields different outcomes: stabilizing two configurations while rendering the other one less stable. Additionally, researchers have explored larger trivacancy (V3) defects within BCC W, where six stable configurations of V3 have been identified [14]. While V3 is a prevalent microvoid in W-PFMs, understanding of the interaction between V3 and He atoms in BCC-W remains limited. For example, the spatial extent to which V3 can capture migrating He atoms is unknown, and the mechanism underlying the structural change in the original V3 defect caused by He atoms at the quantum theory level is still unclear.

Moreover, considering the practical operating environment of W-PFMs within a Tokomak, the plasma inside the reactor reaches an ultrahigh temperature of approximately 10^9^ K. This high-temperature plasma is actually a high-temperature black body that continuously emits a significant quantity of high-energy photons [15,16,17]. When high-energy photons irradiate W-PFMs, they excite some of the electrons within the material, resulting in W-PFMs entering an electronically excited state [17]. The physical behavior of materials in such an electronically excited state can deviate significantly from that observed in the electronic ground state [18,19]. To our knowledge, there is currently a lack of research on V3 defects and their interactions with He in electronically excited states. Undoubtedly, it is essential to uncover the mechanisms behind the observed phenomena in these electronically excited states.

Physically, changes in the microstructure of a system are accompanied by alterations in electronic structures [20,21]. The DFT method provides insights into various physical phenomena from the view of the electronic structure. For example, based on DFT calculations, some researchers have found that the density of states (DOS) at the Fermi level of the He atom in the TIS is lower than that in the OIS, implying that the TIS is a more stable occupation site relative to the OIS [22]. Similar phenomena have also been observed for iron (Fe) [23,24]. Moreover, DFT calculations have revealed a degree of mutual attraction between two interstitial He atoms in materials such as W and other transition metals, leading to the formation of a He pair structure [25,26]. However, it is difficult for current computational resources to support effective calculations of the interactions between complex lattice defects (including microvoids and dislocations) and He atoms. Another widely used theoretical computational method in the scientific community is empirical molecular dynamics (MD). Although empirical MD simulations can handle large-scale lattice defects, they fail to provide insights from an electronic structure perspective. Fortunately, we have developed a tight-binding (TB) potential model for W–He interactions within the framework of quantum theory. This model can handle W systems containing large-scale lattice defects and provide electronic structures for in-depth understanding [27].

In this study, we conducted a thorough investigation into the interaction between He and the most stable V3 configuration in both the electronic ground state and electronically excited states, employing our developed W-He TB potential model [28]. Our TB calculations reveal that the electric dipole interaction between W and He results in the W atom nearest to the He atom on the inner wall of V3 becoming a cation, while the other W atoms on the wall become anions. The attraction between the cation and the anions promotes either the decomposition or conversion of the V3 defect. Furthermore, the high-energy photons emitted from the high-temperature plasma core induce electronic excitation of the defective W system, enhancing the cationic nature of the W atom nearest to the He atom on the V3 wall. Consequently, other W atoms on the V3 wall transition from anions to cations. This transformation caused by electronic excitation plays, to some extent, an inhibitory role in the decomposition and deformation of V3.

## 2. Computational Methods

In this paper, we utilized our developed W-He TB potential model, which has been presented in detail in our previously published work [28], to calculate our systems of interest. Here, we outline the basic spirit of our computational method as follows. In the TB model, the total energy of a system is expressed as the sum of two components [28,29,30]:

(1) $$E_{tot}=E_{band}+E_{rep}.$$

Here, $E_{rep}$ represents the energy associated with repulsion between atoms. $E_{band}$ corresponds to the band structure energy, which is expressed as:

(2) $$E_{band}=2\sum_{m}f_{m}\varepsilon_{m},\;$$

where $\varepsilon_{m}$ denotes the eigenvalue of the m^th^ eigenstate and $f_{m}$ is the Fermi–Dirac distribution function. The factor of 2 arises from two electrons with different spin states.

We assessed the reliability of our developed W-He tight-binding potential model [28]. To achieve this, we calculated the formation energies of a He atom occupying a tetrahedral site ($E_{TIS}^{f}$) and an octahedral site ($E_{OIS}^{f}$) in a perfect 9 × 9 × 9 W cubic supercell in an electronic ground state, which are summarized in Table 1. Clearly, our TB calculations predict formation energies of 6.10 eV and 6.37 eV for He atoms at tetrahedral and octahedral sites, respectively. Earlier DFT calculations suggested formation energies ranging from 6.16 eV to 6.32 eV for a He atom at a tetrahedral site and from 6.38 eV to 6.56 eV for a He atom at an octahedral site [7,31,32,33]. In contrast, calculations based on different classical potentials predict formation energies ranging from 5.67 eV to 6.22 eV for a He atom at a tetrahedral site and from 5.87 eV to 6.39 eV for a He atom at an octahedral site [34,35,36,37]. Therefore, the results from our TB calculations closely align with those from DFT calculations for both tetrahedral and octahedral sites. Additionally, using the climbing image nudged elastic band (CI-NEB) method, we examined the energy barrier for He migration between neighboring tetrahedral sites at the TB level. The obtained migration energy barrier of 0.052 eV is consistent with the 0.06 eV reported in DFT calculations [38]. These benchmarked calculations affirm the reliability of the W-He TB potential model we employed for investigating W-He interactions in this paper.

In this paper, the electron excitation induced by high-energy photons from a high-temperature plasma core within a fusion reactor is considered. Theoretically, intense electronic excitation influences the Hermann–Feynman force acting on each ion within the lattice [39]. Moreover, the relaxation time associated with photon-induced electron excitation and interactions among excited electrons is on the order of femtoseconds (fs), considerably shorter than the relaxation time (on the order of picoseconds (ps)) for electron–phonon interactions. Consequently, the excited electrons reach their equilibrium state before the entire system, including both electrons and the lattice, achieves thermal equilibrium. In an equilibrium state of excited electrons, their distribution in the energetic landscape follows the Fermi–Dirac distribution law [40].

(3) $$f_{m}=\frac{1}{\exp\left[\frac{\varepsilon_{m}-\varepsilon_{F}}{k_{B}T_{e}}\right]+1}.\;$$

Here, $T_{e}$ represents the electronic temperature, which serves as a measure of the electronic thermal equilibrium state, $\varepsilon_{F}$ is the Fermi energy, and $k_{B}$ is the Boltzmann constant.

When the system is in an electronically excited state, its free energy can be expressed as [41]:

(4) $$G=E_{tot}-T_{e}S_{e},$$

where $S_{e}$ denotes the electron entropy addressed as:

(5) $$S_{e}=-2k_{B}\sum_{m}\left[f_{m}\ln f_{m}+\left(1-f_{m}\right)\ln\left(1-f_{m}\right)\right].\;\;$$

## 3. Results and Discussion

### 3.1. Interaction of a Single He Atom with a Trivacancy Defect

#### 3.1.1. The Spatial Range of the Effect of V3 on He

**Figure 1:**
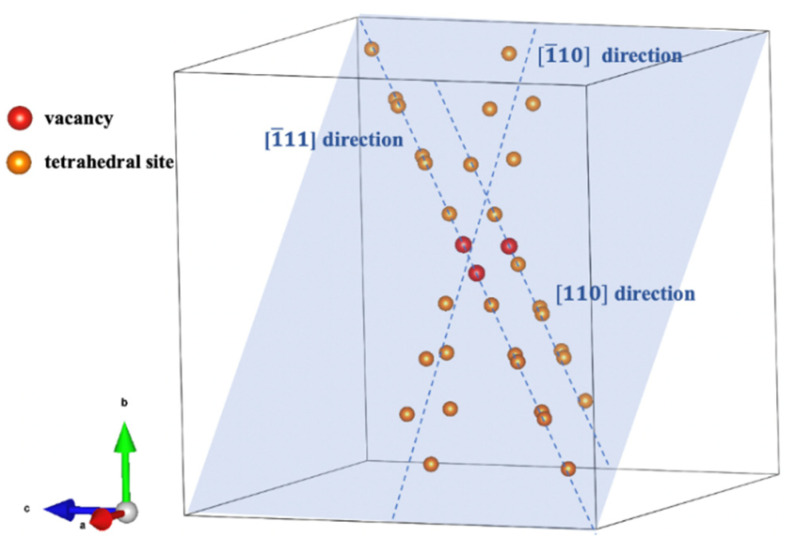
A microvoid consisting of three atomic vacancies (V3) is marked with red dots. The tetrahedral sites at different distances from V3 in three unequal directions (namely, the $\left[\bar{1}11\right]$, $\left[\bar{1}10\right]$, and [110] directions) are marked with orange dots. The W atoms are not shown.

We constructed a 9×9×9 cubic supercell containing 1458 W atoms to simulate a perfect W system. Within this supercell, we created a V3 defect marked with red in Figure 1, which is the most stable configuration among the various V3 configurations. To investigate the interaction between the V3 defect and a He atom, we positioned a He atom at different tetrahedral sites within the supercell, which are denoted with orange dots in Figure 1. These tetrahedral sites are arranged along three unequal directions, namely, the $\left[\bar{1}11\right]$, $\left[\bar{1}10\right]$, and [110] directions, along which different distances between the He atom and the V3 defect are considered. Then, we calculated the trapping energy of a He atom at the different sites described in Figure 1 based on the following equation [42]:

(6) $$E_{He@TriV}^{trap}=\left(E_{He@TriV}-E_{TriV}\right)-\left(E_{He@TIS}-E_{perfectW}\right).$$

Here, $E_{He@TriV}$ represents the total energy of the system with a He atom located at each site mentioned above, and $E_{TriV}$ represents the total energy of the system containing V3 without He. $E_{He@TIS}$ denotes the total energy of the system with a He atom located at a tetrahedral site in the perfect W lattice, and $E_{perfectW}$ is the total energy of the perfect W. The value of $E_{He@TriV}^{trap}$ quantifies the degree of difficulty for a He atom positioned at a tetrahedral site far from V3 to move to a tetrahedral site near V3. If the value of $E_{He@TriV}^{trap}$ is negative, the He atom prefers to stay at the tetrahedral site near V3.

**Figure 2:**
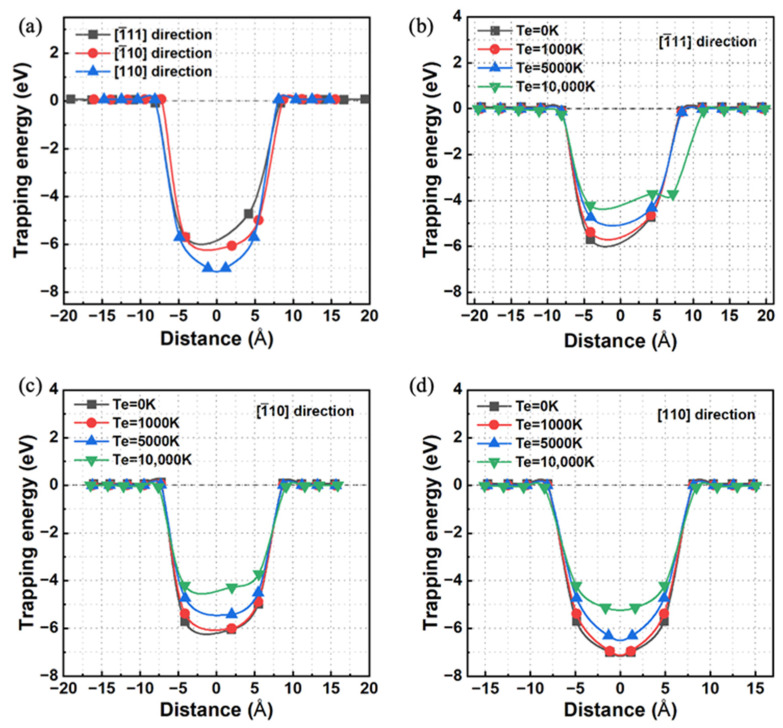
(**a**) In the electronic ground state, the variation in the trapping energy as a function of the distance between the He atom and the V3 defect in three unequal directions ($\left[\bar{1}11\right]$, $\left[\bar{1}10\right]$, and [110] directions). In different electronically excited states, the variation in trapping energy as a function of the distance between the He atom and the V3 defect in the (**b**) $\left[\bar{1}11\right]$, (**c**) $\left[\bar{1}10\right]$, and (**d**) [110] directions.

Figure 2a illustrates the variation in the calculated trapping energy as a function of the distance between the He atom and the V3 defect when the system is in the electronic ground state. Clearly, when the He atom is distributed along the $\left[\bar{1}11\right]$, $\left[\bar{1}10\right]$, or [110] direction, the trapping energies approach zero or become positive once the distance exceeds approximately 8.20 Å, 7.98 Å, and 8.08 Å, respectively. Therefore, the radius of the region around the V3 defect trapping the He atom is estimated to be approximately 8 Å.

Furthermore, the interactions between the V3 defect and the He atom in the electronically excited state were investigated. Here, three different electronically excited states corresponding to electronic temperatures of 1000 K, 5000 K, and 10,000 K are considered. As depicted in Figure 2b–d, when the electronic temperature increases, the region of the V3 defect trapping the He atom in each considered direction slightly expands. Moreover, the trapping energy associated with the He atom located near the V3 defect strikingly increases. This observation indicates that the attractive interaction of the V3 defect with the nearby He atom weakens with increasing electronic excitation.

**Figure 3:**
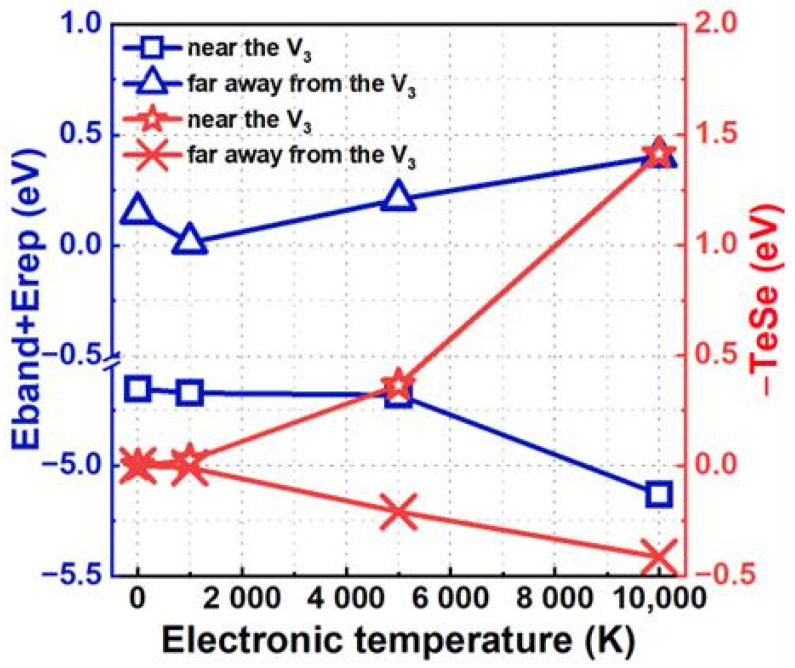
When the He atom is located at the tetrahedron site arranged along the $\left[\bar{1}11\right]$ direction nearest to or far from the V3 defect, the decomposition term of the trapping energy varies with the electronic temperature.

To gain deeper insights into the impact of electronic excitation on the ability of V3 to trap He, we decomposed the trapping energy into three components: band structure energy, repulsive energy, and the energy associated with electron entropy. These components correlate with the electronic structure, atomic structure, and degree of electron disorder distribution within the excited system [28,43]. As illustrated in Figure 3, in the specific scenario where the He atom approaches the V3 defect, the sum of the band structure energy and repulsive energy gradually decreases as the electronic temperature increases. In contrast, the contribution from electron entropy shows a rapid increase. This increased contribution from the electron entropy plays a crucial role in the increase in the V3 trapping energy for He near the V3 defect. Conversely, when the He atom is located far from the V3 defect, the sum of the band structure energy and repulsive energy, together with that contributed from electron entropy, changes slightly when the electronic temperature varies from 0 K to 10,000 K. In these cases, the change in trapping energy arising from the considered electronic excitation can be negligible.

#### 3.1.2. Local Structural Features of the Trivacancy Defect Caused by the He Atom

**Figure 4:**
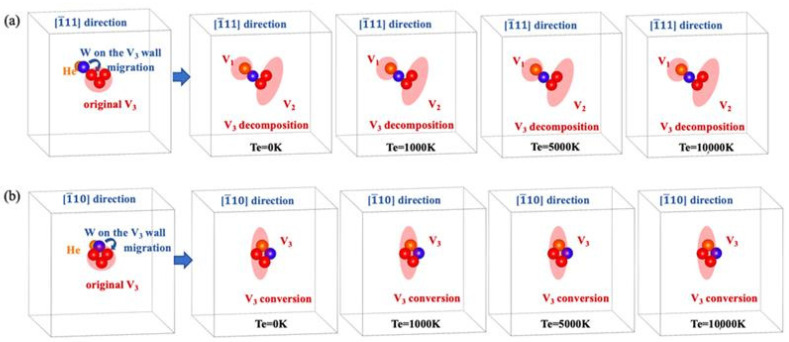
In the electronic ground state and different electronically excited states, (**a**) decomposition of the original V3 occurs when a He atom is located at the tetrahedral site arranged along the $\left[\bar{1}11\right]$ direction nearest to the V3 defect, and (**b**) conversion of the original V3 into another V3 type occurs when a He atom is located at the tetrahedral site arranged along the $\left[\bar{1}10\right]$ direction nearest to the V3 defect.

Now, we carefully examine the local structure around the V3 defect for each case mentioned above. Figure 4a,b shows the detailed structural features around the V3 defect in the electronic ground state and different electronically excited states we considered. As shown in Figure 4a, at different electronic temperatures, when a He atom is located at the tetrahedral site nearest to the V3 defect and arranges along the $\left[\bar{1}11\right]$ direction, the nearest neighboring W atom on the V3 wall migrates into the vacant region of the V3 defect. This leads to the decomposition of the original V3 into a complex defect composed of a monovacancy (V1) and a divacancy (V2). In this case, the He atom is finally trapped by the produced V1. On the other hand, as depicted in Figure 4b, at different electronic temperatures, when the He atom is located at the tetrahedral site nearest to the V3 defect and arranges along the $\left[\bar{1}10\right]$ direction, it pushes the nearest neighboring W atom on the V3 wall toward the center of the V3 defect, causing structural deformation of the original V3 defect.

Similar scenarios emerge when the He atom is located at other sites near V3. Therefore, at different electronic temperatures, the introduction of a He atom near the V3 defect can either catalyze the decomposition of the original V3 into a complex defect composed of V1 and V2 or facilitate the spontaneous conversion of the original V3 into another configuration.

**Figure 5:**
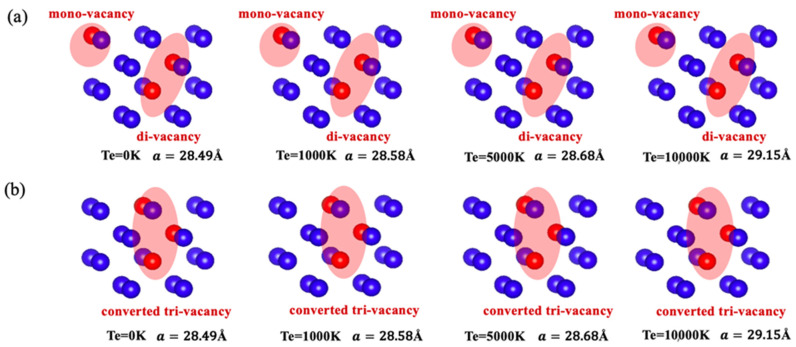
The electronic temperature-dependent evolution of the systems after removing the He atom from the final structures is shown in Figure 4. (**a**) The decomposed V3 structures and (**b**) the converted V3 structure remained unchanged at all considered electronic temperatures.

Next, we investigated whether the decomposed or distorted V3 configurations at different electronic temperatures could return to their original V3 configurations when the introduced He atom was removed. To assess this, we removed the He atom from the decomposed or converted V3 structures, followed by structural optimization for each entire system at the corresponding electronic temperature. As illustrated in Figure 5a,b, the structural characteristics of the decomposed and converted V3 configurations are preserved. This observation indicates that the He-induced decomposed V3 structure and the converted V3 structure are stable even without the presence of He in the electronic ground state and different electronically excited states.

**Figure 6:**
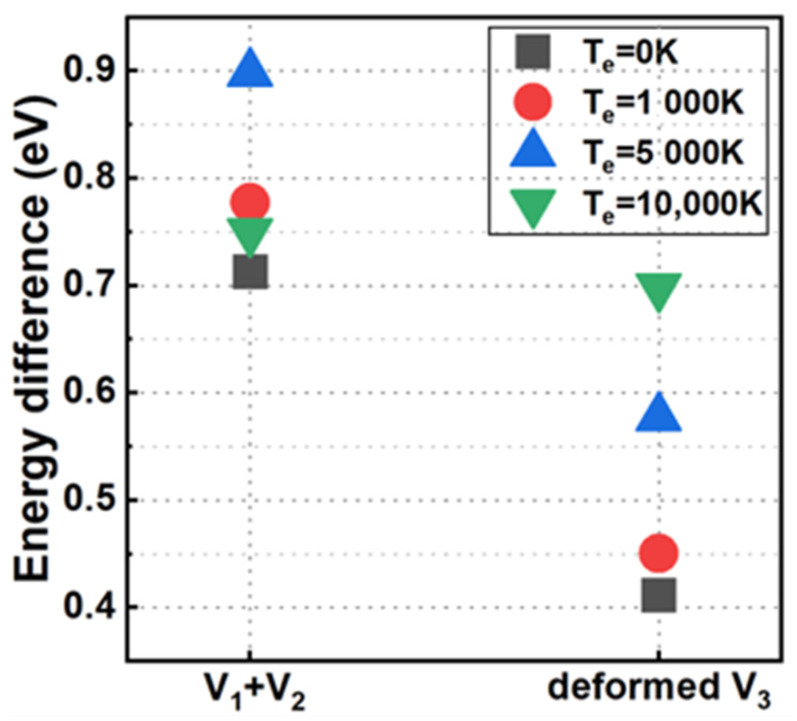
In the electronic ground state and different electronically excited states we considered, the energy differences between the decomposed or converted V3 and the original V3.

Furthermore, we calculated the energy differences between the decomposed or distorted V3 structures mentioned above and the original V3 defect structure in the electronic ground state and different electronically excited states. As depicted in Figure 6, the calculated energy differences are all positive at different electronic temperatures, which implies that the original V3 defect configuration is energetically more stable. Based on these analyses, we believe that the He atom near the V3 defect acts as a catalyst to facilitate the dissociation or distortion of the original V3 defect into metastable structures.

#### 3.1.3. Local Electronic Structure of the Trivacancy Defect Caused by the He Atom

To determine the nature underlying the decomposition and conversion of the original V3 defect caused by the presence of He, we performed an in-depth analysis of the relevant electronic structure of the system in its electronic ground state and various electronically excited states. Here, we calculated the d-electron charges of seventeen W atoms (labeled 1–17) located on the V3 wall in the defective systems at different electronic temperatures. For comparison, the d-electron charges of a W atom in a perfect tungsten system in its electronic ground state are also computed as a baseline. Then, we determined the changes in the d-electron charges of the seventeen W atoms relative to the baseline. If the change in the d-electron charge of a W atom relative to the baseline is positive, it signifies an anionic character; otherwise, it is cationic.

**Figure 7:**
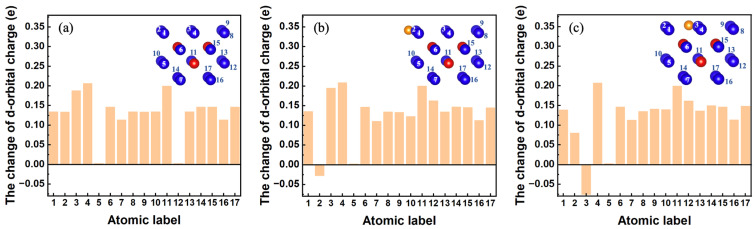
In the electronic ground state, there is a change in the d-orbital charges of seventeen W atoms on the V3 wall. (**a**) Without He. (**b**) When a He atom is located at the tetrahedral site arranged along the $\left[\bar{1}11\right]$ direction nearest to the V3 defect. (**c**) When a He atom is located at the tetrahedral site arranged along the $\left[\bar{1}10\right]$ direction nearest to the V3 defect.

First, we discuss the scenario in the electronic ground state. As displayed in Figure 7a, in the absence of the He atom, the change in the d-electron charge of the seventeen W atoms on the V3 wall is positive, indicating anionic characteristics. However, as shown in Figure 7b,c, when a He atom is located at a nearby tetrahedral site arranged along the $\left[\bar{1}11\right]$ or $\left[\bar{1}10\right]$ direction, the changes in the d-electron charges of its nearest **#**2 or **#**3 W atoms on the V3 wall decrease to negative values, exhibiting cationic characteristics. Nevertheless, the changes in the d-electron charges of other W atoms on the V3 wall remain positive, showing anionic characteristics. Based on these calculations, we can infer that the presence of the He atom induces some electron charge transfer among certain W atoms. Consequently, electrostatic attractions occur between the **#**2 or **#**3 W cation and other W anions on the V3 wall. This electrostatic attraction, in turn, facilitates the migration of the **#**2 or **#**3 W atom into the vacant region of V3. Therefore, in the electronic ground state, the change in the local electronic structure of V3 caused by the He atom, which is closest to the V3 defect, promotes the decomposition of the original V3 or its conversion into another configuration of V3.

**Figure 8:**
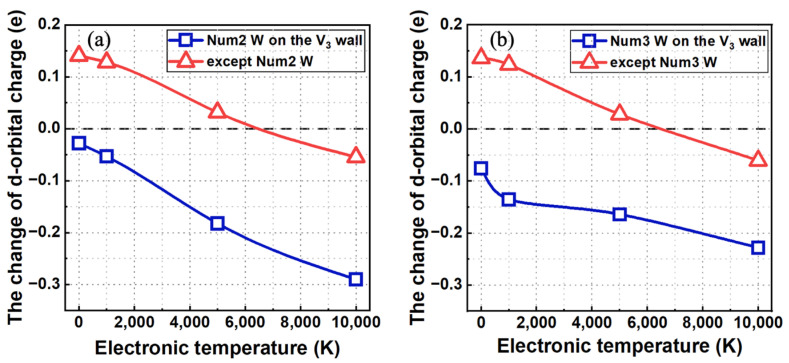
When a He atom is located at the tetrahedral site arranged along (**a**) the $\left[\bar{1}11\right]$ direction or (**b**) the $\left[\bar{1}10\right]$ direction nearest to the V3 defect, the change in d-electron charges of (**a**) the #2 W atom and other W atoms on the V3 wall or (**b**) the #3 W atom and other W atoms on the V3 wall as a function of electronic temperature.

Furthermore, we estimated the changes in the d-electron charges of the seventeen W atoms on the V3 defect wall relative to the above baseline at different electronically excited states. As shown in Figure 8a, when a He atom is located at the tetrahedral site arranged along the $\left[\bar{1}11\right]$ direction, with increasing electronic temperature, the change in the d-electron charge of the **#2** W atom remains negative and displays a downward trend. This trend implies an increase in the cationic nature of the **#**2 W atom as the electronic temperature increases. Interestingly, the change in the average d-electron charges of other W atoms on the V3 defect wall transitions from positive to negative at an electronic temperature of 10,000 K. This means that these W atoms exhibit anionic characteristics at electronic temperatures of 0 K and 5000 K but transition to cationic characteristics when the electronic temperature is 10,000 K. Accordingly, the electrostatic interaction between the **#**2 W atom and other W atoms on the V3 defect wall is attractive within the electronic temperature range of 0 K–5000 K. However, it becomes repulsive at 10,000 K. A similar phenomenon is also observed when a He atom is located at the tetrahedral site arranged along the $\left[\bar{1}10\right]$ direction, as depicted in Figure 8b. Therefore, the stronger the electronic excitation of the system, the stronger the ionization characteristics of W atoms on the V3 wall. The electrostatic interactions between the W atom on the V3 walls nearest to the He atom and other W atoms on the V3 wall gradually transition from attraction to repulsion. The change in the electronic structure caused by electron excitation can indeed hinder the W atom on the V3 wall closest to the He atom from entering the vacancy region of V3. Therefore, electronic excitation plays an inhibitory role in the deformation or dissociation of V3.

### 3.2. Behavior of a Trivacancy Defect Trapping He Atoms

We sequentially introduced more He atoms into the V3 defect until the number of He atoms inside the V3 defect reached eight. When each He atom was added, the atomic structure of the entire system was sufficiently relaxed at each corresponding electronic temperature. Upon careful examination of the resulting structures, we observed that although eight He atoms are present inside the original V3 defect, the overall structural shape of the original V3 remains almost the same.

Furthermore, we calculated the trapping energy of the He atoms as follows:

(7) $$E_{He_{n}@TriV}^{trap}=\left(E_{He_{n}@TriV}-E_{He_{n-1}@TriV}\right)-\left(E_{He@TIS}-E_{perfectW}\right).$$

**Figure 9:**
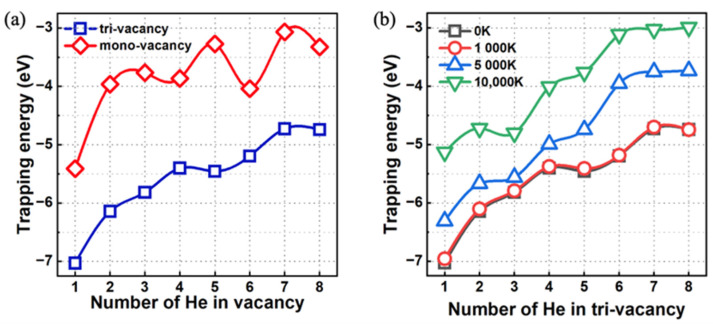
(**a**) Trapping energies of 1–8 He atoms inside the monovacancy and trivacancy states in the electronic ground state. (**b**) Trapping energies of 1–8 He atoms inside the trivacancy in different electronically excited states measured at different electronic temperatures.

Figure 9a shows the trapping energy as a function of the number of He atoms in the monovacancy or trivacancy state at the electronic ground state. The trapping energy evidently increases as the number of trapped He atoms in either the V3 defect or the monovacancy increases in the electronic ground state, where the computed trapping energy of He within the V3 defect is significantly lower than that within a monovacancy. This phenomenon indicates that V3 defects possess a greater capacity to trap He atoms than do monovacancy defects. We further evaluated the trapping energy in electronically excited states measured at different electronic temperatures of 1000 K, 5000 K, and 100,000 K. As shown in Figure 9b, the trapping energy gradually increases with increasing electronic temperature, except that the energy curve for 1000 K is almost the same as that for the ground state (0 K). This observation indicates that the ability of V3 defects to trap He atoms weakens with increasing electronic temperature.

**Figure 10:**
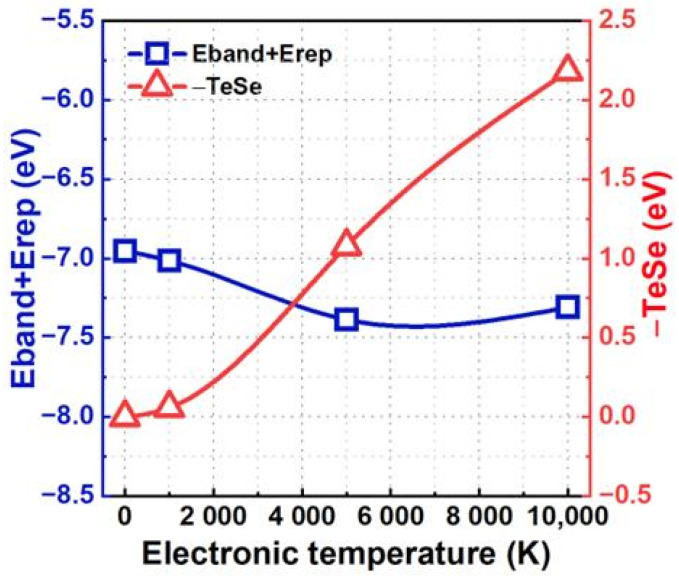
The decomposition terms of the trapping energy vary with the electronic temperature when the trivacancy contains one He atom.

We also decompose the trapping energy into three components: band structure energy, repulsion energy, and electron entropy [28,43]. As depicted in Figure 10, the sum of the band structure energy and the repulsive energy slightly decreases with increasing electronic temperature. In contrast, the energy associated with electron entropy increases rapidly. This suggests that the predominant factor driving the increase in trapping energy is the enhanced electron disorder distribution resulting from electronic excitation.

## 4. Conclusions

In this paper, we propose that the radius of the region around the V3 defect trapping the He atom is approximately 8 Å. Furthermore, we offer a comprehensive understanding of the physical mechanism underlying He-induced decomposition and the conversion of the original V3 by analyzing the electronic structures. We found that the presence of a He atom near the V3 defect along the $\left[\bar{1}11\right]$ or $\left[\bar{1}10\right]$ direction induces significant changes from −0.135 e to +0.025 e or from −0.135 e to +0.075 e for the W atom closest to the He. Moreover, the other W atoms on the V3 wall transition to anions. This cationic W atom interacts with other W anions on the inner wall of V3, promoting V3 decomposition or conversion in its structure. Moreover, the increase in electronic excitation enhances the cationic characteristics of the W atom closest to the He atom and prompts other W atoms on the V3 wall to change from anions to cations. Consequently, this transition alters the electrostatic interaction from attraction to repulsion between them, which hinders the W atom on the V3 wall nearest to the He atom from entering the vacant region within V3. Therefore, electronic excitation plays a certain inhibitory role in the decomposition and deformation of V3.

We emphasize that during the working period of a nuclear fusion reactor, as neutron-induced damage accumulates in W, more He atoms become trapped by vacancies. Simultaneously, the formation of neutron-induced defects enhances the retention of He and enlarges the size of the He nanobubbles. The interaction radius between He and a vacancy defect in W is a fundamental factor underlying these phenomena. Furthermore, understanding the evolution of the microstructure induced by He atoms at different electronic excited states provides profound insights into the performance of W-PFMs in nuclear fusion reactors.

## Figures and Tables

**Table 1:** Formation energy of a He atom in a tetrahedral site ($E_{TIS}^{f}$) or an octahedral site ($E_{OIS}^{f}$). Energy barrier of a He atom migrating between adjacent tetrahedral sites ($E_{TIS}^{mig}$). The unit of energy is eV.

	Li ^a^	Chen ^b^	Justin ^c^	Bonny1 ^d^	Bonny2 ^d^	TB	DFT
$E_{TIS}^{f}$	6.21	6.22	6.16	5.67	5.79	6.10	6.32 ^e^, 6.23 ^f^, 6.16 ^g^, 6.22 ^h^
$E_{OIS}^{f}$	6.39	6.34	6.31	5.87	6.03	6.37	6.56 ^e^, 6.48 ^f^, 6.38 ^g^, 6.44 ^h^
$E_{TIS}^{mig}$	—	—	—	—	—	0.052	0.06 ^i^

^a^ Ref. [35]; ^b^ Ref. [34]; ^c^ Ref. [36]; ^d^ Ref. [37]; ^e^ Ref. [7]; ^f^ Ref. [31]; ^g^ Ref. [33]; ^h^ Ref. [32]; ^i^ Ref. [38].

## Data Availability

The raw data supporting the conclusions of this article will be made available by the authors upon request.
